# Making better CRISPR libraries

**DOI:** 10.7554/eLife.21863

**Published:** 2016-11-03

**Authors:** Shiyou Zhu, Wensheng Wei

**Affiliations:** 1 Biodynamic Optical Imaging Center, State Key Laboratory of Protein and Plant Gene Research, School of Life Sciences, Peking University, Beijing, China; 1 Biodynamic Optical Imaging Center, State Key Laboratory of Protein and Plant Gene Research, School of Life Sciences, Peking University, Beijing, Chinawswei@pku.edu.cn; 2 Peking University-Tsinghua University-National Institute of Biological Sciences Joint Graduate Program, Peking University, Beijing, China; 3 Beijing Advanced Innovation Center for Genomics, Peking University, Beijing, China; 4 Peking-Tsinghua Center for Life Sciences, Peking University, Beijing, China

**Keywords:** CRISPR, genetic screening, nucleosomes, Human

## Abstract

A new algorithm improves the performance of CRISPR-based genetic screens in mammals.

**Related research article** Horlbeck MA, Gilbert LA, Villalta JE, Adamson B, Pak RA, Chen Y, Fields AP, Park CY, Corn JE, Kampmann M, Weissman JS. 2016. Compact and highly active next-generation libraries for CRISPR-mediated gene repression and activation. *eLife* **5**:e19760. doi: 10.7554/eLife.19760

Since the human genome sequence was completed in 2003, genome-wide screening has become a popular method for quickly associating specific genes with their roles in cells. More recently, the CRISPR-Cas9 system has become the dominant tool for genome-editing (Jinek et al., 2012; Cong et al., 2013; Mali et al., 2013) and it has subsequently been adapted to make highly effective genetic screening platforms (Shalem et al., 2014; Zhou et al., 2014).

The CRISPR-Cas9 system is derived from the methods used by certain bacteria to identify and cut up foreign genetic material (Barrangou et al., 2007). To edit the genome, specially designed RNA molecules guide a nuclease enzyme called Cas9 to the location of interest in the DNA sequence; the Cas9 enzyme then cuts the DNA at this position. A mutant form of Cas9 that is unable to cut DNA can also be used to generate libraries of single guide RNAs (sgRNAs) that target regions around transcription start sites in the genome. By allowing researchers to either repress or activate gene expression – techniques that are known as CRISPR interference (CRISPRi) and CRISPR activation (CRISPRa), respectively – these sgRNAs make it possible to carry out powerful genetic screens in mammalian cells (Gilbert et al., 2014; Konermann et al., 2015). Now, in eLife, Jonathan Weissman and colleagues at the University of California, San Francisco – including Max Horlbeck as first author – report that a new algorithm can predict the activity of sgRNAs more accurately than existing algorithms (Horlbeck et al., 2016a).

Many factors affect the ability of sgRNAs to activate or repress genes including the sequence, length and secondary structure of the sgRNA (Doench et al., 2014; Xu et al., 2015). Furthermore, the DNA in mammalian cells (and also in other eukaryotic cells) is packaged inside structures called nucleosomes, which make it difficult for the Cas9 enzyme to access the DNA (Hinz et al., 2015; Horlbeck et al., 2016b; Isaac et al., 2016). This is particularly important for CRISPRi and CRISPRa screens because the mutant Cas9 enzyme must stay bound to the DNA for extended periods of time. Horlbeck et al. therefore optimized the design of their sgRNAs to target DNA regions that were not packaged in nucleosomes and thus were more accessible to mutant Cas9.

To improve the CRISPRi and CRISPRa libraries that they had made previously (Gilbert et al., 2014), Horlbeck et al. analyzed data from 30 CRISPRi screens and 9 CRISPRa screens and defined “activity scores” for every sgRNA relative to the sgRNA with the strongest activity for each gene. They then used this information to make new CRISPRi and CRISPRa libraries that contained the ten most active sgRNAs for each gene.

The new human CRISPRi library was used to screen chronic myeloid leukemia K562 cells to identify genes that are essential for cell growth. Impressively, this library was able to identify about 10% more essential genes compared with the original CRISPRi library (Gilbert et al., 2014). Furthermore, a half-sized version of the new human CRISPRi library (with only the top five sgRNAs per gene) performed similarly to the full-sized version. This is reassuring because smaller libraries are easier to construct and use in screens. Similarly, Horlbeck et al. also demonstrated that the new human CRISPRa library outperformed the original one.

Horlbeck et al. found that, when used with the mutant form of Cas9, none of the CRISPRi libraries had toxic side effects like those observed with other approaches that use the active enzyme (Wang et al., 2015). This makes it possible to effectively identify genes, even if they show only slight differences in expression compared to negative controls.

To summarize, this study established an effective algorithm to predict the activity of sgRNAs based on the location of nucleosomes in the genome. Horlbeck et al. used this algorithm to generate new CRISPRi and CRISPRa libraries with much improved performance in genetic screens in humans and mice. It remains to be seen if the algorithm could be used to optimize other types of CRISPR screens, especially ones that use the normal Cas9 enzyme.
